# Do Poor Functional Outcomes and Higher Morbidity Following Emergency Repair of Giant Hiatus Hernia Warrant Elective Surgery in Asymptomatic Patients?

**DOI:** 10.3389/fsurg.2021.628477

**Published:** 2021-02-11

**Authors:** Iulia Bujoreanu, Daniya Abrar, Savvas Lampridis, Ravindra Date

**Affiliations:** ^1^General Surgery Department, Royal Preston Hospital, Preston, United Kingdom; ^2^Thoracic Surgery Department, 424 General Military Hospital, Thessaloniki, Greece; ^3^Department of Cancer, The University of Manchester, Manchester, United Kingdom

**Keywords:** emergency, paraesophageal hernia, giant hiatus hernia, morbidity, mortality

## Abstract

**Background:** Patients with a giant hiatus hernia may present with acute symptoms caused by obstruction, strangulation, perforation and uncontrolled bleeding. Emergency surgical repair has been associated with significant mortality and even greater morbidity. The aim of this study is to investigate the short-term outcomes following emergency repair of giant hiatus hernias.

**Methods:** Data were retrospectively collected for all patients who underwent emergency surgical repair of giant hiatus hernia in a university teaching hospital between 2009 and 2019. Outcomes were short-term morbidity and mortality. We also assessed the association of clinical predictor covariates, including age, ASA class and time to surgery, with risk for major morbidity.

**Results:** Thirty-seven patients with a median age of 68 years were identified. Following surgery, 9 patients (24.3%) developed organ dysfunction that required admission to the intensive care unit. Two patients (5.4%) underwent revision surgery and 3 (8.1%) developed pneumothorax that necessitated chest drain insertion. The commonest complication was pneumonia, which occurred in 13 patients (35.1%). Two deaths (5.4%) occurred within 30 days from surgery.

**Conclusions:** Emergency repair of giant hiatus hernia is associated with high rates of major morbidity, which includes poor functional status, further interventions, repeat surgery, and admission to the intensive care unit. Larger studies are warranted for long-term follow-up to assess post-operative quality of life is needed for asymptomatic patients and for those undergoing emergency surgery.

## Introduction

Since centralization of Upper Gastro-Intestinal cancer services in 2009, exponential growth in referrals for oesophago-gastric emergencies such as esophageal perforations and incarcerated giant hiatus hernias (GHH) was noted. This tertiary referral unit maintains prospective data of all the giant hernias (>30% stomach in chest), that undergo elective as well as emergency surgery.

GHH comprise 5–10% of all hiatus hernias (1) and mainly affects elderly population (2, 3). Current guidelines recommend elective surgery for symptomatic hernias and watchful wait for asymptomatic ones (4, 5). Acute complications requiring emergency repair include gastric volvulus, uncontrolled bleeding, obstruction, strangulation, and perforation (2, 6). The risk of developing acute symptoms that necessitate emergency surgery is estimated between 0.7 and 7% (7). Despite this relatively low incidence, clinicians must be mindful of these patients, as the mortality rate of emergency surgery ranges from 7 to 50%, which is much higher than that of elective repair (0.65%) (8, 9). Patients who require an emergency operation are also subject to 5-fold increased odds of serious morbidity compared with patients undergoing elective surgery (8).

Aim of this study is to present the short-term outcomes of non-elective GHH repair in a single institution over a 10-year period, review the pertinent literature and provide insights into decisions for asymptomatic patients.

## Patients and Methods

### Patient Selection

Eligible for inclusion in this study were patients who underwent non-elective repair of GHH at Royal Preston Hospital, United Kingdom after 1 January 2009. Non-elective surgery was defined as urgent (patient requiring admission for symptom management, with hernia repair during the same admission) or emergency (immediate operation in a patient with acute complications).

### Data Extraction

Two authors (DA, IB) retrospectively reviewed the medical records of the identified patients. The same authors independently extracted data using a predefined form and then crosschecked them. The collected variables included patient characteristics (age, sex, presenting symptoms, type of paraoesophageal hernia, and Charlson comorbidity index), endoscopic and imaging investigations, operative data [American Society of Anaesthesiologists (ASA) class, surgical approach and technique] and outcome data over a 30-day period (higher level of care, complications, length of hospital stay and mortality). Higher level of care included level 2 (patients requiring more detailed observation or intervention, including support for a single failing organ system) and level 3 (patients requiring advanced respiratory support alone, or monitoring and support for two or more organ systems). The Charlson comorbidity index was used to report on preoperative morbidity because it has been shown to be a valid and reliable method to measure disease burden and has been widely utilized in clinical research (10, 11). A commonly used version of this index identifies 17 comorbidities that are associated with mortality and appropriately assigns weights to each of them, ranging from 1 to 6 points. The final score is obtained via the summation of applicable points and ranges from 0 (no disease burden) to 29 (maximal disease burden) (12). We used the revised Clavien-Dindo classification to grade postoperative complications, as it is has been validated in emergency surgical patients (13, 14). A major complication was defined as a Clavien-Dindo grade III or IV, indicating the need for an intervention or a life-threatening complication requiring admission to the intensive care unit.

### Statistical Analysis

Descriptive statistics were summarized with frequencies and percentages for categorical variables. Data were calculated as mean ± standard deviation for continuous variables; when the distribution was skewed, median and interquartile range (IQR) were reported instead. Major morbidity was defined as Clavien-Dindo grade III or IV. Predictor variables were defined as age, ASA class and time to surgery. The threshold of statistical significance was set at a *P*-value of < 0.05. Statistical analysis was performed using SPSS Statistics 26 (IBM Corp., Armonk, NY).

## Results

A total of 42 consecutive patients underwent emergency or urgent repair of GHH between January 2009 and October 2019. Of these, five patients were excluded because their medical records were not available for review. The demographics, baseline clinical characteristics and presenting symptoms of the included patients are summarized in Table 1. Transfers from other hospitals accounted for 46% of admissions. Diagnostic investigations included computed tomography (CT) in 34 patients (91.9%), oesophagogastroduodenoscopy (OGD) in 11 (29.7%) and barium swallow imaging in 3 (8.1%). The surgical management of the patients is outlined in Table 2.

**Table 1:** Demographic, clinical, and preoperative characteristics of the patients.

Sex—no. (%)	
Male	18 (48.6)
Female	19 (51.4)
Median age—years	68 (IQR, 65–81)
Type of hernia—no. (%)	
II	1 (2.7)
III	20 (54)
IV	16 (43.3)
Comorbid conditions—no. (%)	
Cardiac disease^a^	13 (35.1)
Pulmonary disease^b^	9 (24.3)
Glomerular filtration rate <30%	1 (2.7)
Anticoagulation therapy	1 (2.7)
Charlson comorbidity index^c^	
Median	3
Mode	4
Range	0–7
Presenting symptoms—no. (%)	
Vomiting	27 (73)
Abdominal pain	21 (56.8)
Constipation	11 (29.7)
Chest pain	6 (16.2)
Dysphagia	6 (16.2)
Peritonism	1 (2.7)
ASA class^d^–no. (%)	
II	17 (46)
III	13 (35.1)
IV	6 (16.2)
V	1 (2.7)

a *Cardiac disease includes coronary artery disease, heart failure, severe valve disease, and significant arrhythmia*.

b *Pulmonary disease includes chronic obstructive pulmonary disease and interstitial lung disease*.

c *The Charlson Comorbidity Index quantifies an individual's burden of disease; the higher the score, the more likely the predicted outcome will result in mortality or higher resource use*.

d *The American Society of Anaesthesiologists (ASA) physical status classification system assesses a patient's pre-anesthesia medical comorbidities, with higher classes indicating more severe systemic disease and class V indicating a moribund patient who is not expected to survive without the operation*.

*IQR, interquartile range*.

**Table 2:** Surgical management of patients.

Time of surgery—no. (%)	
Day	29 (78.4)
Night	8 (21.6)
Median time to surgery—days	3 (IQR, 1–5.5; range, 0–31)
Surgical approach—no. (%)	
Laparoscopy	16 (43.3)
Laparoscopy converted to laparotomy	6 (16.2)
Laparotomy	14 (37.8)
Robotic	1 (2.7)
Surgical technique—no. (%)	
Hiatal repair	30 (81)
Fundoplication	16 (43.3)
Nissen fundoplication	5
Anterior fundoplication	6
Posterior fundoplication	1
Not specified	4
Gastropexy	15 (40.5)
Mesh repair	2 (5.4)
Gastrectomy	1 (2.7)

*IQR, interquartile range*.

The surgical outcomes are detailed in Table 3. The commonest postoperative complication was pneumonia, occurring in 13 patients (35.1%). Seven patients (18.9%) required total parenteral nutrition, 3 (8.1%) developed pneumothorax that necessitated chest drain insertion, and 2 patients (5.4%) had new-onset atrial fibrillation that was treated with correction of electrolyte disturbances. There were no recorded cases of postoperative myocardial infarction, pulmonary embolism, or cerebrovascular accident.

**Table 3:** Surgical outcomes.

Higher level of care^a^–no. (%)	31 (83.7)
Cardiovascular support—no. (%)	11 (29.7)
Mechanical ventilation—no. (%)	4 (10.8)
Median length of stay—days	1 (IQR, 0–2; range, 0–10)
Clavien-Dindo classification^b^–no. (%)	
Grade I	11 (29.7)
Grade II	11 (29.7)
Grade III-a	3 (8.1)
Grade III-b	1 (2.7)
Grade IV-a	5 (13.5)
Grade IV-b	4 (10.8)
Grade V	2 (5.4)
Median length of hospital-stay—days	12.5 (IQR, 6.3–18.6; range, 4–38)

a *Higher level of care includes level 2 (patients requiring more detailed observation or intervention, including support for a single failing organ system or postoperative care) and level 3 (patients requiring advanced respiratory support alone, or monitoring and support for two or more organ systems)*.

b *The Clavien-Dindo classification categorizes complications as grade I for any deviation from the normal postoperative course, grade II for those requiring pharmacological treatment, grade III for those requiring intervention (grade III-b if under general anesthesia), and grade IV for those requiring intensive care unit management (grade IV-b for multiorgan dysfunction)*.

*IQR, interquartile range*.

Two deaths occurred in the patient cohort. The first patient was an 86-year-old man with a Charlson comorbidity index of 7 who was transferred from another hospital with sepsis and symptoms of gastrointestinal obstruction. He underwent open repair and died on the fourth postoperative day from multiorgan failure. The second patient was an 84-year-old man with a Charlson comorbidity index of 6 who presented with coffee ground vomiting and was transferred from another hospital. Eleven days following open repair, he developed peritonitis secondary to perforation of the transverse colon and underwent a further open repair. He died 7 days later.

Two patients who had very poor functional outcomes despite adequate restoration of anatomy and are discussed here.

A 51-year-old man presented with epigastric pain and vomiting. On the third day of admission, he underwent laparoscopic repair of a herniated gastric fundus through a large crural defect. He subsequently developed progressive dysphagia, and a barium swallow investigation revealed a long esophageal stricture which was considered to be of peptic etiology. This required multiple endoscopic balloon dilations. A follow-up barium swallow also showed narrowing at the level of the pylorus, suggestive of gastric outlet obstruction. He has been monitored with regular outpatient clinic appointments.

A 78-year-old lady presented with profuse coffee-ground vomiting secondary to GHH and underwent laparoscopic repair on the fourth day of admission. She was found to have almost complete herniation of the stomach with partial volvulus, and a gastrostomy tube was inserted. Postoperatively, she developed intractable vomiting. Investigations revealed no evidence of gastric outlet obstruction but there was significant delay in gastric emptying, suggestive of gastroparesis. As no clinical improvement was noted, a decision was made to use a jejunal extension of the gastrostomy to maintain nutritional requirements. The patient died 3 years later from an unrelated cause.

## Discussion

Whereas long-term outcomes in emergency GHH repairs have been thoroughly discussed, short-term outcomes have been less well studied. Compared to the available literature, the recurrence rate was low (5.4%, *n* = 2) and mortality was acceptable (5.4%, *n* = 2). Mortality for acute presentations is higher when compared to our previous published data for elective laparoscopic repairs of GHH (2.9%) (15, 16). However, morbidity was high (35%) and some of the complications were unusual and difficult to explain.

Probably due to the advanced age, most of our patients had comorbid conditions, with more than half suffering from a cardiorespiratory disease. Similar were the findings in other studies, in which patients presenting acutely were older and had higher prevalence of at least one cardiopulmonary comorbidity compared to those undergoing elective GHH repair (17–19). In our study, the median Charlson comorbidity index score was 3 (range, 0–7). Tam et al. (17) reported an age-adjusted Charlson comorbidity index score of 4 (range, 0–6) for 171 patients undergoing non-elective repair of paraesophageal hernia. In contrast, the same score was significantly lower (2; range, 0–4; *p* < 0.0001) for 753 patients who underwent elective repair.

Advanced age and comorbidity, along with urgency of surgery, have all been postulated to predict patient-specific risk of postoperative morbidity and mortality. Ballian et al. (20) assessed 980 patients who underwent GHH repair (80% elective and 97% laparoscopic) and developed clinical prediction models for in-hospital or 30-day mortality and major morbidity using logistic regression. A 4-covariate prediction model consisting of age >80 years, pulmonary disease, congestive heart failure and urgency of operation provided discriminatory accuracy for postoperative mortality of 88%. A 5-covariate model, which further included sex, was 68% predictive of major postoperative morbidity. Urgency of operation was a significant predictor of major morbidity (non-elective 41% vs. elective 18%; *P* < 0.001) and mortality (non-elective 8% vs. elective 1.1%; *P* < 0.001). Markar et al. (21) investigated the influence of hospital volume upon clinical outcomes by retrospectively reviewing 12,441 cases of paraesophageal hernia causing obstruction or gangrene. High hospital volume was an independent predictor of reduced 30-day mortality (odds ratio 0.65, 95% confidence interval 0.55–0.76, *P* < 0.001). In addition, multivariate regression analysis showed significant factors associated with 30-day mortality included age >70 years, chronic congestive cardiac failure, ischemic heart disease, dementia, as well as pulmonary and renal disease. In this study, the logistic regression model that was performed to ascertain the effects of age, ASA class and time to surgery on major morbidity was not statistically significant (*P* = 0.18).

Several studies published over the last two decades have reported on surgical outcomes following non-elective repair of GHH. Table 4 presents the results of such studies, with the exclusion of registry-based studies, those that did not report on emergency data separately and studies with <10 patients. Complication rates varied significantly, ranging from 28 to 100%. Major postoperative morbidity (Clavien-Dindo grades III or IV complications) ranged from 8 to 30%. In our study, this rate was slightly higher at 35%. Recurrence rates ranged between 7 and 18%. In the present study, recurrence occurred only in 2 patients (5.4%), both of which underwent revision surgery. The mortality of emergency paraesophageal hernia repair varied between 0 and 22%.

**Table 4:** Surgical outcomes of large studies reporting on emergency repair of paraesophageal hernia published in the last two decades.

**First author**	**Year**	***n***	**Complications**	**Clavien-Dindo grade I–II**	**Clavien-Dindo grade III–IV**	**Recurrence**	**Reoperation**	**Mortality**
Quinn (22)	2019	51	NR	NR	5 (10%)	5 (10%)	NR	7
Shea (23)	2019	30	14 (47%)	7 (23%)	4 (14%)	5 (17%)	0	3 (10%)
Sorial (24)	2018	18	6 (28%)	1 (6%)	5 (28%)	NR	NR	0
Wirsching (19)	2018	38	11 (29%)	11 (29%)	0	NR	0	0
Tam (17)	2017	171	65 (38%)	NR	NR	2 (7%)	20 (12%)	8 (5%)
Light (25)	2016	29	29 (100%)	NR	NR	4 (14%)	NR	4 (14%)
Shaikh (26)	2013	25	12 (48%)	NR	NR	3 (12%)	NR	5 (20%)
Polomsky (18)	2010	23	17 (74%)	10 (44%)	7 (30%)	NR	3 (13%)	5 (22%)
Rogers (27)	2001	13	NR	NR	5 (8%)	NR	NR	1 (8%)
Geha (28)	2000	20	NR	NR	NR	2 (10%)	NR	2 (10%)
Bujoreanu	2020	37	19 (51%)	8 (22%)	15 (40%)	2 (5.4%)	2 (5.4%)	2 (5.4%)

*n, number of patients; NR, not reported*.

In our unit we also follow-up patients with GHH who either decline surgery or are deemed unsuitable for operative management. Follow-up is augmented by the Quality of Life in Reflux and Dyspepsia (QOLRAD) questionnaire for the first 2 years. Since 2014, a total of 17 such patients were followed up. Of these, four decided to have surgery at a later date and only one presented acutely with volvulus. Longer follow-up times might reveal a higher number of patients who ultimately need surgery.

The main limitations of the present study include the retrospective collection of data and relatively small number of patients, probably because of rarity of condition itself. Furthermore, unlike the standardized surgical approach in elective surgery, there is variability in the emergency situation. While operating on high risk, elderly and frail patients it may be necessary to adopt a minimalistic approach and only perform gastropexy or gastrostomy to prevent volvulus. In our unit we attempt complete excision of the sac followed by crural repair and fundoplication. However, this is not always possible in high-risk patients leading to heterogeneity in surgical technique.

## Conclusions

Patients admitted with acute GHH symptoms are older and have more comorbid conditions compared to their counterparts who undergo elective surgical repair. Major postoperative complications, which require intervention, repeat surgery or admission to the intensive care unit, may occur in as many as one in three patients. Proceeding to surgery should be thoroughly discussed with these patients as surgery may save their life but leave them with major morbidity. Operating on these patients in the elective setting might avoid high morbidity and mortality. Larger studies to investigate prediction models for post-operative complications will allow risk stratification and informed management decisions.

## Data Availability Statement

The original contributions presented in the study are included in the article/supplementary materials, further inquiries can be directed to the corresponding author.

## Ethics Statement

Ethical review and approval was not required for the study on human participants in accordance with the local legislation and institutional requirements. Written informed consent for participation was not required for this study in accordance with the national legislation and the institutional requirements.

## Author Contributions

IB: literature review and writing up of article. DA: data collection. SL: writing up of article. RD: concept, supervision, and review of final article. All authors contributed to the article and approved the submitted version.

## Conflict of Interest

The authors declare that the research was conducted in the absence of any commercial or financial relationships that could be construed as a potential conflict of interest.
